# The prognostic value of NIRS in preterm infants with (suspected) late-onset sepsis in relation to long term outcome: A pilot study

**DOI:** 10.1371/journal.pone.0220044

**Published:** 2019-07-24

**Authors:** Inge A. Zonnenberg, Jennifer van Dijk, Frank A. M. van den Dungen, R. Jeroen Vermeulen, Mirjam M. van Weissenbruch

**Affiliations:** 1 Department of Neonatology, Emma Children’s Hospital, Amsterdam UMC, Vrije Universiteit Amsterdam, Amsterdam, The Netherlands; 2 Department of Medical Psychology, Emma Children’s Hospital, Amsterdam UMC, Vrije Universiteit Amsterdam, Amsterdam, The Netherlands; 3 Department of Child Neurology, Neuroscience Campus Amsterdam, Emma Children’s Hospital, Amsterdam UMC, Vrije Universiteit Amsterdam, Amsterdam, The Netherlands; Hopital Robert Debre, FRANCE

## Abstract

Late-onset sepsis is frequently seen in preterm infants and is associated with poor neurodevelopmental outcome. White matter damage is proposed as substrate of poor outcome, with contributing factors as regional hypoxia and effects of cytokines on oligodendrocytes. We investigated the relation between cerebral oxygenation during (suspected) late-onset sepsis and neurodevelopmental outcome. Prospective cohort study, including preterm infants (gestational age <32 weeks and/or birthweight <1500 grams) with (suspected) late-onset sepsis underwent NIRS registration during the first 72 hours of suspected late-onset sepsis. At two years corrected age neurodevelopment was scored using the Bayley Scales of Infant Development-II. Thirty-two infants were included. Twenty-seven infants were identified with proven late-onset sepsis and five infants had clinical sepsis without positive blood culture. In this study, late-onset sepsis was predominantly caused by coagulase negative staphylococci (CoNS) (72%). All NIRS values were within normal limits. No association was found between NIRS and impaired neurodevelopmental outcome (n = 4) at corrected age two years: composite cognitive score 105 (80–115), composite motor score 103 (82–118) (median and range). In this pilot study, late-onset sepsis (predominantly caused by CoNS with a relatively mild clinical course), was not associated with aberrant NIRS values, nor with impaired neurodevelopmental outcome. Further research might establish our findings and elucidate effects of other micro-organisms on cerebral perfusion.

## Introduction

Preterm and very low birth weight infants are at risk of acquiring nosocomial sepsis due to their immature immune system[1]. Approximately 21–36% of the admitted infants experience a culture proven late-onset sepsis. Up to 50% of the infants was treated with antibiotics during their NICU stay, both for blood culture proven and clinically suspected infection [2, 3].

The long-term effects of sepsis in preterm newborns are well established. Sepsis is associated with adverse long term neurodevelopment, with mental or psychomotor developmental delay[3]. However, also studies have been published, concerning mainly perinatal CoNS infections, in which this relation has not been established[4].

The adverse long term effects of sepsis are linked to the development of white matter injury. [5, 6], and white matter injury is associated with abnormal psychomotor outcome[3, 7]. One of the mechanisms inducing white matter injury is compromised cerebral blood flow and thus hypoxemia, which can be the result of dysregulation of vital parameters like blood pressure, due to infection. Another mechanism is release of inflammatory mediators, which can induce and increase cerebral damage[8, 9].

To evaluate effects of nosocomial infection on the brain in preterm infants, cerebral imaging by cerebral ultrasounds or MRI can be performed. However, cerebral ultrasound will only show already established gross brain damage. Other modalities of brain monitoring might provide an additional value, e.g. by detecting changes in cerebral perfusion, which may prelude cerebral damage.

Several studies have determined reference values of regional cerebral oxygen saturation in preterm infants [10–12]. Also studies concerning the association between regional cerebral oxygen saturation and long term outcome become more and more available. However, most studies report the regional cerebral oxygenation during the first 72 hours of life. Alderliesten *et al*. showed an association between lower regional cerebral oxygen saturation in the first 72 hours of life and lower neurodevelopmental outcome scores[13]. In another follow-up study, this association could not be demonstrated[14].

Van der Laan *et al*. studied multisite fractional tissue oxygen extraction during an episode of clinical sepsis on short term outcome[15]. The effects on cerebral oxygenation during late-onset sepsis, and in relation to long term neurodevelopmental outcome, have not been described previously. In search for a causal link, it would be interesting to investigate whether impairments in cerebral autoregulation might be of influence in development of white matter injury and relate to subsequent long term development. One could hypothesize cerebral oxygenation is compromised during late-onset sepsis due to instability of the infant during the first days after onset and thereby influence long term outcome.

Aim of this study was to investigate the feasibility to study if late-onset sepsis effects the cerebral oxygenation during the first 72 hours after onset and its relation with psychomotor outcome at corrected age of two years.

## Materials and methods

We performed a prospective, observational cohort study: the INFANT study. Data of preterm infants < 32 weeks gestational age and/or < 1500 grams admitted to the level III Neonatal Intensive Care Unit (NICU) of the VU Medical Center between August 2012 and December 2014 and suspected of late-onset sepsis were prospectively collected. Preterm infants with syndromal or chromosomal abnormalities and congenital metabolic disorders were excluded. Informed parental consent was obtained and approval was given by the medical ethical committee of the VU University Medical Center (protocol number 2008/77).

Late-onset sepsis was suspected when one of the following clinical symptoms occurred: hypothermia (<36,5 °C) or hyperthermia (>37,5 °C), hypotension, tachycardia, apnea, feeding problems, irritability and/or apathy. Late-onset sepsis was defined as a positive blood culture after 72 hours of life[16, 17]. When blood culture remained sterile, but antibiotic treatment was given for seven days due to persistent clinical symptoms, late-onset sepsis was considered probable and defined as clinical sepsis.

Clinical data were collected from patients’ medical charts. Neonatal variables included gestational age at birth, birth weight, gender, intraventricular hemorrhage according to Volpe[18] and white matter echogenicity on cerebral ultrasound defined as any white matter hyperechogenicity occurring during the study period on any localization in the cerebrum, need for inotropic support, need for mechanical ventilation and lumbar puncture.

During the first 72 hours of suspicion of late-onset sepsis, cerebral oxygenation was registered by INVOS 5100C near infrared spectrometer (Covidien/ Medtronic, Boulder, Colorado, USA) in combination with the small adult sensor Somasensors with emitter-detector distance of 3 and 4 cm (Covidien/ Medtronic) with data storage every 34 seconds. The sensor was placed left or right frontoparietal on the patients head providing regional cerebral oxygenation (rScO_2_) and replaced every three hours to prevent skin lesions. Change of position of the sensor might alter the rScO2 value measured, however Hyttel-Sorensen *et al*. state out of range values on average will tend to normalize[19]. Isolated drop out data after repositioning of the sensor were removed. Arterial oxygen saturation (SaO2) was retrieved by pulse oximetry on a limb. To investigate the balance between oxygen delivery and oxygen consumption, the relative cerebral fractional tissue oxygen extraction (cFTOE) can be formulated as a ratio: (SaO_2_-rScO_2_)/ SaO_2_. Analyses were performed in time frames of eight-hour duration. Artifacts in registrations were assessed. If a value of zero was registered, this value was eliminated from the data set except for severely respiratory or circulatory compromised patients, which could correspond with a value of zero. All other values were maintained.

Cognitive and motor development was scored at two years corrected age by the Bayley Scales of Infant Development II (BSID-II), consisting of a mental developmental index, psychomotor developmental index and a behavioral rating scale [20]. The outcome was rated good when BSID-II >85 and poor if BSID-II<85 or non-survived. Also language skills were assessed by the lexi quotient[21, 22].

Statistical analysis was performed with SPSS using version 22 (SPSS Inc, Chicago, Illinois, USA). For multivariate analysis we used the non-parametric Kruskal Wallis test and if appropriate the Mann-Whitney U test. Due to the non-normality of the data these non-parametric tests were chosen. Mixed models were used as appropriate for longitudinal data analysis. A probability P value < 0.05 was considered statistically significant. To obtain a difference of 15 points (one standard deviation) on the rating scale of BSID-II with a type I error of 5% and a power of 18% 32 patients were needed for this study.

## Results

During the study period, 32 infants (14 boys, 18 girls) were included. Patient characteristics are shown in Table 1 and presenting clinical symptoms are stated in Table 2. Twenty-seven infants were identified with proven late-onset sepsis and five infants had clinical symptoms, elevated CRP and were treated with antibiotics for seven days, but without positive blood culture. Three infants experienced two episodes of late-onset sepsis suspicion. Only the first episode was used for analysis. CoNS (n = 23) were the causal agents in 72% in cases with proven late-onset sepsis. *Staphylococcus aureus* (n = 3) and *Escherichia coli* (n = 1) counted for the other proven late-onset sepsis episodes.

**Table 1:** Patient characteristics.

Gender: boys, n (%)	14 (44%)
Gestational age, weeks (range)	27 4/7 (24 0/7–32 0/7)
Birth weight, grams (range)	1029 (545–1900)
Survival, n (%)	31 (97%)
Postnatal day of onset late-onset sepsis	10 (4–48)
Lumbar puncture, n (%)	23 (72%)
Need for intubation during LOS	8 (25%)
Need for inotropic support	2 (6%)
Signs of NEC stage II/III during episode of LOS	2 (6%)
White matter abnormalities on cerebral ultrasound	4 (13%)

LOS: late-onset sepsis, gestational age and birthweight stated as median and range

**Table 2:** Presenting clinical symptoms.

Apnea/ increased need of oxygen	24 (75%)
Apathy	19 (59%)
Tachycardia	13 (41%)
Temperature-instability	11 (34%)
Feeding problems	10 (31%)
Hyperglycemia	2 (6%)
Hypotension	1 (3%)

**Fig 1:**
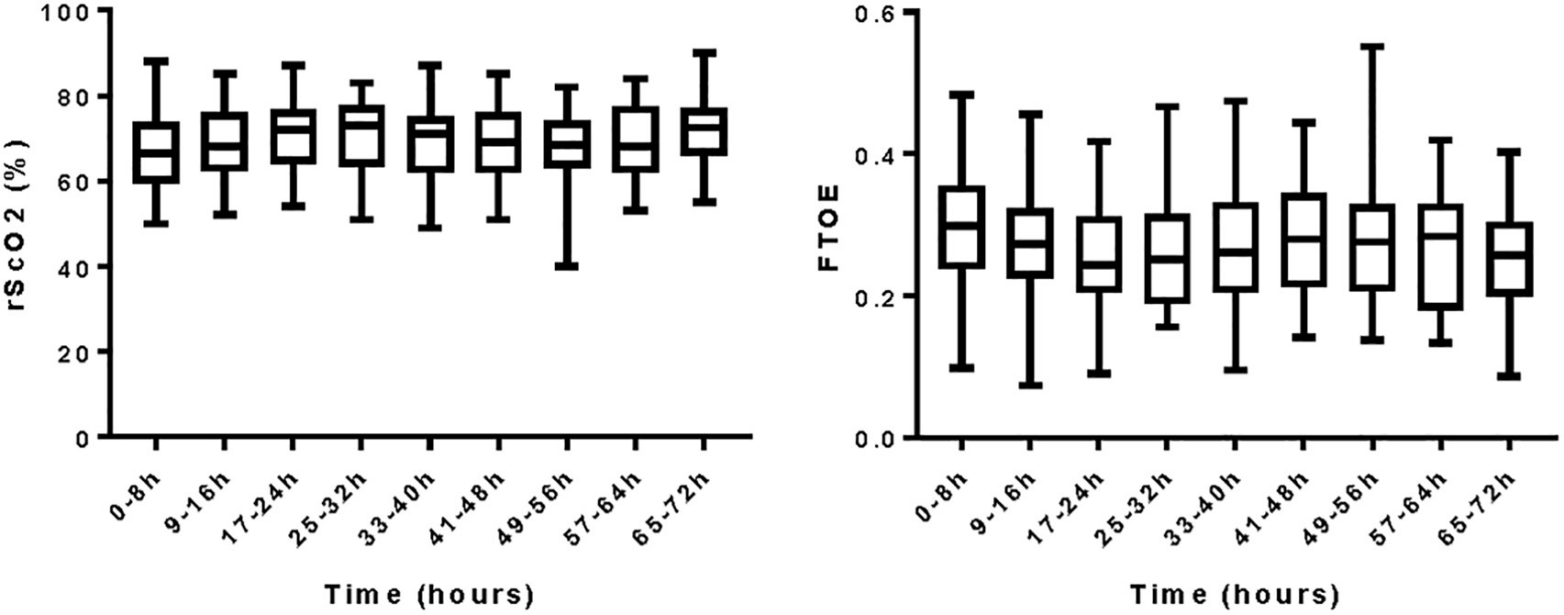
Mean rScO2 and FTOE during first three days of late-onset sepsis. rScO_2_: regional cerebral oxygenation saturation; cFTOE: cerebral fractional tissue oxygen extraction; h: hours.

Fig 1 shows the mean values of rScO2 and cFTOE in the first 72 hours of late-onset sepsis. The median postnatal day of onset late-onset sepsis was day 10 (range day 4 to 48). Longitudinal analysis shows no significant difference over the 72-hour registration, either for rScO2 nor FTOE (respectively p = 0.536 and p = 0.588).

**Fig 2:**
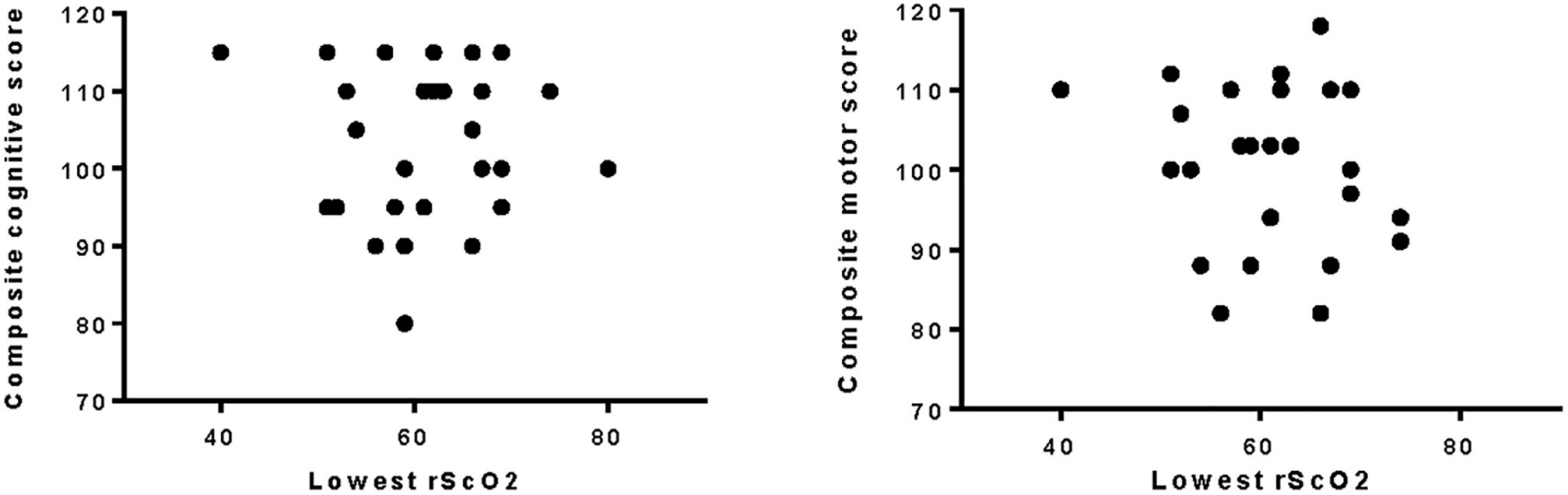
Lowest rScO2 (8 hour interval) and composite cognitive and motor outcome based on the Bayleys Scales of Infant Development II. rScO_2_: regional cerebral oxygenation saturation.

Cognitive and motor development was assessed at corrected age of two years. One patient of 32 patients died during NICU admittance. Twenty-eight of 31 patients who survived were tested (90%). Three patients were lost to follow-up due to them moving abroad (1), cancellation of the test due to personal circumstances (1) and one patient turned out to have a severe structural cerebral disorder (double cortex) for which this patient was excluded for follow up analysis. Results of the median cognitive and motor development were within normal range (Table 3). Though one patient scored under 85 for cognitive development (score 80) and two patients below 85 for motor development (score for both patients 82). Also lexi quotient and total behavioral scores are within normal range. There was no correlation between lowest mean value of rScO2 during the 72 hour period and the composite cognitive score (Fig 2).

**Table 3:** Median corrected age, cognitive score, motor score, total behavioral score and lexi quotient.

Corrected age at testing (range)	24m 4d (23m 3d–31m 0d)
Corrected age composite cognitive score (range)	105 (80–115)
Corrected age composite motor score (range)	103 (82–118)
Total behavioral score (range)	21 (3–56)
Lexi quotient (range)	92 (61–119)

m: months; d: days

Brain ultrasound abnormalities occurred in four patients. rScO2 and FTOE in the first 72 hours of late-onset sepsis were not associated with echo densities on cerebral ultrasound in any localization in the cerebrum, nor with good outcome (BSID-II score mental and motor ≥ -1 SD) nor with poor outcome (BSID-II either mental or motor < -1SD, or non-survived).

## Discussion

During the first 72 hours of late-onset sepsis we did not detected changes in cerebral tissue oxygenation and consumption as measured by NIRS in our study cohort of preterm infants in comparison to the reference values the first days after birth as presented by Alderliesten *et al*[10] as best available reference values at this moment. Other studies report data regarding cerebral oxygenation in subsequent days after birth showing a small decline in rScO2 values, however these studies had small study populations[11]. Due to possible small alterations in cerebral perfusion during late-onset sepsis which could not be demonstrated in this study population, a larger cohort is required to elucidate the effect of late-onset sepsis on cerebral perfusion. Also, to assess the association of regional cerebral oxygen saturation and long term neurodevelopmental outcome larger cohorts are needed[19].

In this cohort CoNS were highly represented, 72% of all proven infections, which is in line with previously published results[23]. CoNS infections are known to have a relatively mild clinical presentation and are not associated with poor motor outcome[4]. In line with the relatively good prognosis of CoNS infections we did not find brain damage in terms of white matter abnormalities in this cohort using cerebral ultrasound[24]. We could not establish an effect on cerebral oxygenation. The latter might be explained by cerebral autoregulation not being compromised during CoNS sepsis. In addition, with NIRS monitoring cerebral oxygenation is measured more superficially including the cortex, but excluding a major part of the white matter. Only two infants needed inotropic support suggesting that the cerebral tissue oxygenation is compliant enough to warrant appropriate cerebral oxygenation in these infants.

The association of white matter injury and decreased values of rScO2 has been described in infants with congenital heart disease[25]. It is also important to consider that an increase of cFTOE ratio might either indicate a reduced oxygen delivery to the brain with a constant oxygen consumption of the brain, or a higher oxygen consumption than delivery[26] and a possible indicator for ominous white matter injury due to regional hypoxia. In this study cerebral ultrasound has been used as a modality of imaging, which is available in the acute period of infection, and might index patients at risk for white matter injury. When exploring sequelae of cerebral hypoperfusion in terms of white matter abnormalities on ultrasound no significant differences were found in rScO2 or cFTOE. One could speculate whether the lowest value of cerebral oxygenation is the most predictive of white matter injury, or of the burden of cerebral hypoxia is a more accurate predictor of white matter damage and thereby of more predictive value for long term psychomotor outcome.

In contrast to most findings, Alshaikh *et al*. reported that CoNS infections were associated with a cognitive delay at 36 months corrected age[27]. However, we found in this cohort the BSID-II at 24 months corrected age median composite scores for cognitive and motor development were in line with the general population. Three individual patients scored less than 85 for either cognitive or motor development at the BSID-II test. Also when exploring the lowest rScO2 values during the infectious episode no relation could be demonstrated with cognitive or motor outcome. Taken into account it is a relatively small study cohort smaller differences between the CoNS group and the general population might have remained undetected, and therefore the relation between CoNS, rScO2, and adversed long term outcome cannot be rejected. Also, follow-up at school age might provide more insight in more subtle cognitive disturbances due to late-onset sepsis. On the other hand, it might also be the representation of a relatively mild course of a CoNS sepsis. Further studies should be performed to determine if the hypothesis if “disturbed cerebral oxygenation might be related to adversed long term outcome in CoNS sepsis” can be proven or should be rejected.

In our cohort we also explored the relation between rScO2 and cFTOE and a good (BSID-II score mental and motor ≥ -1SD) or a poor outcome (BSID-II either mental or motor < -1SD, or non-survived). We were not able to show any relation between NIRS values in the first 72 hours during late onset infection and neurodevelopmental outcome at two years. However, as CoNS infections are the most frequent etiology of late-onset sepsis, it would be interesting to explore the effects of micro-organisms other than CoNS in a larger cohort than this study provides.

We do acknowledge limitations of our study. This is a single center study in a relatively small group of preterm infants. It would be interesting to study a larger cohort and compare the cerebral oxygenation values and long term follow up to a control group and evaluate other factors influencing cerebral oxygenation and the effects on long term outcome. Also, if a larger cohort is available the changes in cerebral oxygenation can be studied in more detail and confounding effects, of interventions in respiratory and circulatory support for instance, could be addressed more appropriately.

Furthermore, the high incidence of coagulase-negative staphylococci limited the number of patients with late-onset sepsis caused by other bacteria. Therefore, it is difficult to assess if cerebral tissue oxygenation is influenced differently during late-onset sepsis caused by different micro-organisms like gram-negative bacteria. And if so, if there is any difference in disturbance of cerebral tissue oxygenation in the relatively mild course of CoNS versus gram negative bacteria. This pilot study could not address differences of effects on long term outcome between groups of patients with CoNS and gram negative micro-organisms, although different effects could be expected due to the more clinical instability during gram negative septicemia.

NIRS monitoring registers the mixed oxygen saturation from superficial tissue e.g. the cerebral cortex and part of the white matter. Also, it is not certain whether white matter and deep grey matter were equally well represented in the registered values of cerebral oxygenation [28]. Therefore, conclusion concerning the relation between white matter abnormalities and NIRS measured oxygen consumption should be drawn with care.

With this study we explored possibilities for future research in terms of combining cerebral oxygenation and cerebral ultrasound in the first days of infection in relation to causing micro-organisms. It would be very interesting to model these findings with other changes during infection, for example the changes in inflammatory mediators. In future studies these limitations have to be addressed with a larger study population to be able to identify the possible effects of these imaginable contributing factors.

In conclusion, in this pilot study we did not detect any changes in cerebral oxygenation during the first 72 hours of late-onset sepsis. In this small cohort we did not detect an effect of late-onset sepsis on neurodevelopmental outcome at two years corrected age. However, this might be the result of a relatively mild course of late-onset sepsis by CoNS. Further research could elucidate the effect of other causal micro-organisms.

## Abbreviations

cFTOE: Cerebral fractional tissue oxygen extraction
CoNS: Coagulase-negative staphylococci
CRP: C-reactive protein
HS-PDA: Hemodynamic significant persistent ductus arteriosus
NEC: Necrotizing enterocolitis
NICU: Neonatal Intensive Care Unit
NIRS: Near-Infrared Spectroscopy
rScO_2_: Regional cerebral oxygen saturation
SaO2: Arterial oxygen saturation
SD: Standard deviation
